# Combining functional magnetic resonance imaging with transcranial electrical stimulation

**DOI:** 10.3389/fnhum.2013.00435

**Published:** 2013-08-05

**Authors:** Catarina Saiote, Zsolt Turi, Walter Paulus, Andrea Antal

**Affiliations:** Clinic for Clinical Neurophysiology, Universitätsmedizin, Georg-August-UniversitätGöttingen, Germany

**Keywords:** non-invasive brain stimulation, transcranial direct current stimulation (tDCS), transcranial random noise stimulation (tRNS), fMRI, transcranial electrical stimulation (tES), neuromodulation

## Abstract

Transcranial electrical stimulation (tES) is a neuromodulatory method with promising potential for basic research and as a therapeutic tool. The most explored type of tES is transcranial direct current stimulation (tDCS), but also transcranial alternating current stimulation (tACS) and transcranial random noise stimulation (tRNS) have been shown to affect cortical excitability, behavioral performance and brain activity. Although providing indirect measure of brain activity, functional magnetic resonance imaging (fMRI) can tell us more about the global effects of stimulation in the whole brain and what is more, on how it modulates functional interactions between brain regions, complementing what is known from electrophysiological methods such as measurement of motor evoked potentials. With this review, we aim to present the studies that have combined these techniques, the current approaches and discuss the results obtained so far.

## Introduction

**Figure 1:**
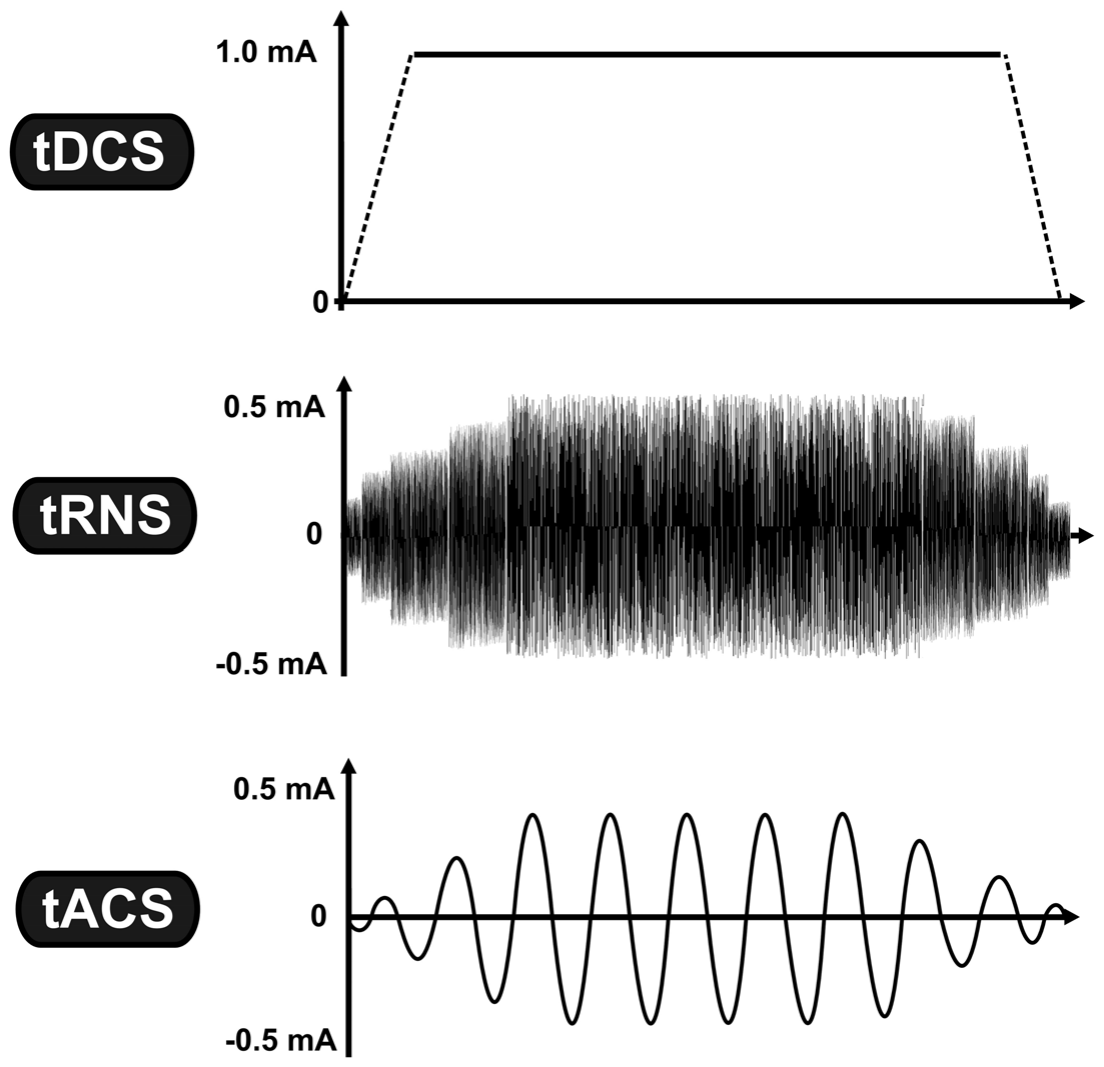
**Transcranial electrical stimulation is a specific subgroup of non-invasive brain stimulation techniques, which is based on the application of low-intensity electrical current.** While tDCS uses constant current intensity, tRNS and tACS use oscillating current. The vertical axis represents the current intensity in milliamp (mA), while the horizontal axis illustrates the time-course. Abbreviations: tDCS, transcranial direct current stimulation; tRNS, transcranial random noise stimulation; tACS, transcranial alternating current stimulation.

Non-invasive brain stimulation (NiBS) techniques use externally applied stimulation for inducing neuroplastic changes in the human brain. Sub-threshold transcranial electrical stimulation (tES) is a specific subgroup of NiBS using low-intensity electrical current (usually between 0.4 and 2.0 mA) via two conductive electrodes placed on the scalp (Nitsche et al., 2008). Despite a considerable shunting effect (Miranda et al., 2006), a certain proportion of the weak electrical current penetrates the scalp and causes prolonged but reversible changes in the cortical excitability by modifying spontaneous neural activity of the neurons (Bindman et al., 1964). One of the most studied tES techniques is transcranial direct current stimulation (tDCS),which is based on the application of a constant current (Nitsche and Paulus, 2000), whereas other tES techniques utilize oscillating currents in a various frequency range [(e.g., from 0.1 to 5000 Hz) (for a review see Paulus, 2011)]. Regarding the application of oscillating current we currently have two approaches: In the case of transcranial random noise stimulation (tRNS), several frequencies are applied within a normally distributed frequency spectrum (between 0.1 and 100 Hz for low-frequency tRNS and 101 and 640 Hz for high-frequency tRNS) (Terney et al., 2008), whereas in transcranial alternating current stimulation (tACS) a single sinusoidal - the most common—waveform at a specific frequency (e.g., at 20 Hz) is given (see Figure 1) (Antal et al., 2008).

The after-effects of tES dominantly depend on the stimulation parameters, including the stimulation duration, the current intensity, the electrode size, the current density (current intensity/electrode size) (Faria et al., 2011), the type of current (direct, oscillating current, or their combination), additional factors related to the current type (e.g., stimulation frequency in the case of oscillating current) (Antal et al., 2008), the timing of the stimulation (e.g., before, during, or after task performance) (Pirulli et al., 2013), and the electrode montage (i.e., position of the electrodes) (Bikson et al., 2012). It is important to notice however, that other tES-independent factors could also potentially influence the outcome of stimulation, such as the wakefulness of the participants (Huber et al., 2013), the state of participants receiving the stimulation (e.g., during rest or during behavioral/cognitive performance) (Silvanto et al., 2008), the individual differences in the neuroanatomy of the brain, genetic polimorphismus (e.g., Brain derived neurothrophic factor; BDNF) (Antal et al., 2010), handedness (Schade et al., 2012), and the experimental paradigm (e.g., motor, visual, cognitive). The relative contribution of each factor is less clear due to the fact that most of the studies apply remarkably different stimulation parameters and the lack of the studies systematically manipulating each factor while controlling the other parameters.

## tES techniques and fMRI

The after-effects induced by sub-threshold tES have been first demonstrated on physiological and behavioral studies (for a review see Stagg and Nitsche, 2011). Transcranial magnetic stimulation (TMS) has been the most commonly used method for evaluating the after-effects of tES on the motor cortex. It is able to detect changes in cortical excitability and depending on the TMS protocol, it can provide information about the influence of tES on aspects of cortico-cortical and cortico-spinal excitability, intracortical inhibition, and facilitation as well as inter-hemispheric interactions (Nitsche et al., 2005). However, this method does not provide information about multifocal brain activation or neural network properties that essentially influence the outcome of stimulation. Functional magnetic resonance imaging (fMRI) has the advantage of providing whole brain data with high spatial precision and with a relatively high temporal resolution in a safe and non-invasive way. Offers a wide range of possibilities for analyzing brain activity and can, therefore, contribute to further elucidate the effects of tES. Previous studies have demonstrated that transcranial application of electrical currents over the motor cortex with supra-threshold intensity induces local and distant BOLD responses in motor related areas (Brandt et al., 1996; Brocke et al., 2008). Nevertheless, these studies were performed using significantly higher current intensities, and we will focus on sub-threshold tES.

**Figure 2:**
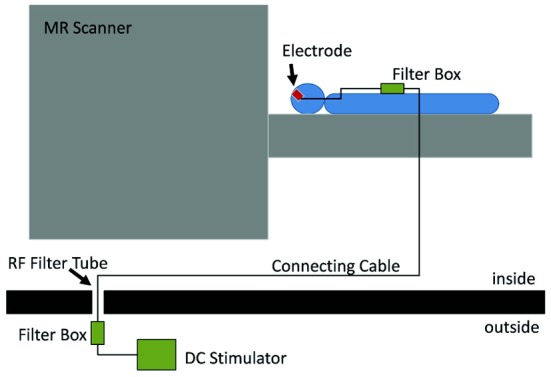
**Experimental setup which allows stimulation inside the scanner and also concurrently with fMRI.** The electrodes on the subjects' head are connected to a filter box, itself connect by a cable going through the wall of the scanner room. This cable is connected to another filter box, to which the stimulator is connected. Reprinted from Antal et al. (2011), with permission from Elsevier.

The joint application of tES and fMRI was initially prevented by the technical difficulties regarding both safety of the procedure and quality of the acquired data (see Figure 2 for a typical setup). The main safety concern is the possibility of heating under the electrodes due to the radio-frequency pulses of the scanner (Lemieux et al., 1997). To prevent this, electrodes wires have been equipped with resistors close to the electrodes. When stimulation is not performed during image acquisition, one has to consider the effect of the stimulation equipment in image quality. This has been shown to cause only a small (3 and 8%) reduction in signal-to-noise ratio (SNR) (Antal et al., 2011) and no distortion in the structural or functional images when electrode cables were unplugged from the stimulator (Polanía et al., 2011). Even though the changes in SNR remain minimal for simultaneous imaging and stimulation (Antal et al., 2011), it is possible to detect artifacts caused by the stimulation. Mild susceptibility artifacts not reaching brain tissue were detected under a frontal electrode (Antal et al., 2011), and B0 field distortions were as well restricted to the scalp (Holland et al., 2011). In a recent study, the artifacts induced by tDCS on functional images were investigated in 2 post-mortem subjects (Antal et al., 2012a,b) In accordance with previous observations, highest artifacts were found in the scalp and in the cerebrospinal fluid (CSF) at the surface and in the ventricles. However, it is relevant to note that the magnitude of the tDCS induced effect was found to be comparable (approximately ½) to that of a physiological BOLD response during finger-tapping using the same imaging sequence. This must be taken into account when interpreting results from concurrent tDCS and fMRI studies. Nevertheless, the technical advances that overcame these difficulties in the last years, have led to an increase in the number of studies combining these tES and fMRI.

The aim of the present article is to review these recent findings about the effect of low-intensity tES on the motor and cognitive functions accompanied by the related brain activity.

## Modulation of activation elicited by motor tasks

**Figure 3:**
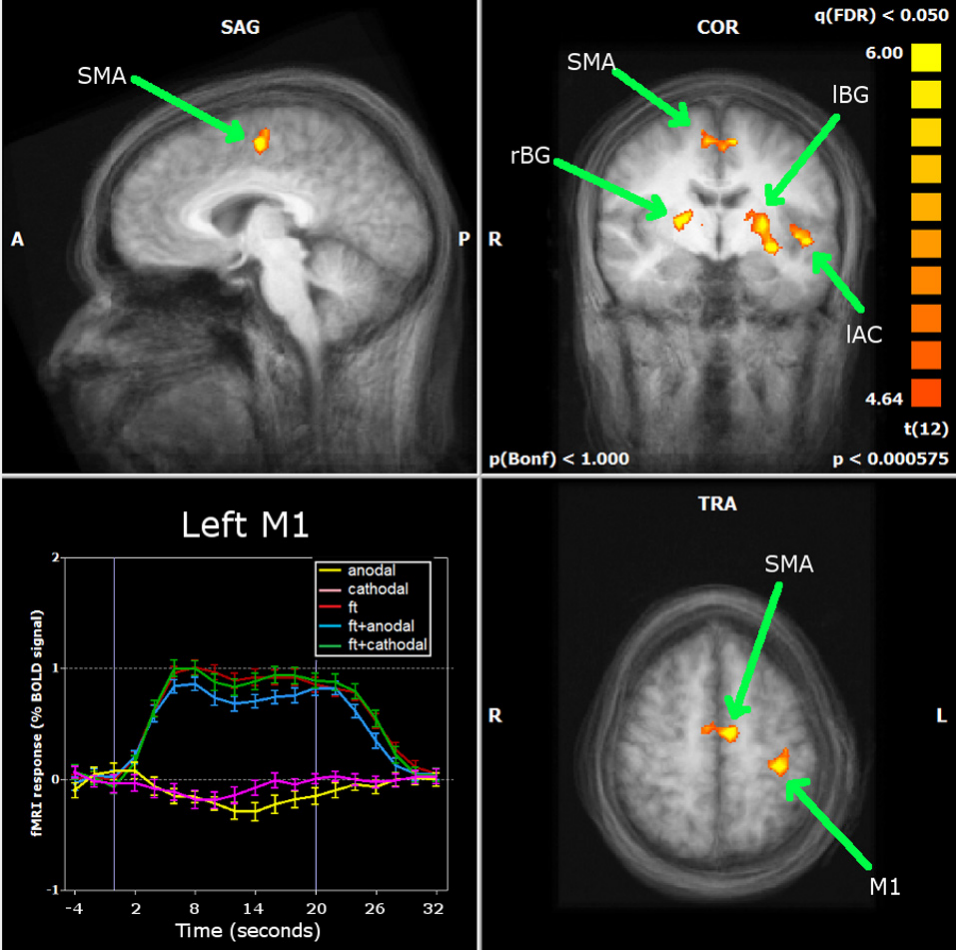
**Antal et al. (2011) carried out a ROI analysis on the regions identified by whole brain analysis during finger movements: primary motor cortex (M1), supplementary motor area (SMA), left and right basal ganglia (lBG and rBG) and left auditory cortex (lAC).** Neither stimulation alone nor combined with finger movements induced significant changes in the BOLD response of the M1. Only in the SMA was a significant decrease found during anodal stimulation simultaneously with finger movements. Reprinted from Antal et al. (2011), with permission from Elsevier.

The large majority of early tDCS studies targeted the motor cortex. Likewise, the first attempts to characterize the effects of the stimulation using fMRI focused on motor related brain areas. Baudewig et al. (2001) compared the activation maps elicited by sequential right hand finger movements before and after 5 min of stimulation. Stimulation was delivered during rest, with one electrode place over the hand area of the left primary motor cortex (M1) and the other over the contralateral supraorbital region(CSR) (left M1-CSR montage), at the intensity of 1 mA. The only significant finding was that cathodal tDCS over the M1 reduced the extent of activation in the supplementary motor area (SMA)—an effect still noticeable 15 min after the end of stimulation—suggesting that a reduction in excitability associated with tDCS was accompanied by a reduction in brain activity. In this study, no changes in activation were found in the M1 as an after-effect of stimulation, and the same was described when analyzing how motor-related activation was modulated during tDCS (Figure 2) (Antal et al., 2011). In this study, using a block design alternating periods of 20 s rest and tDCS during a finger tapping task, a decrease in activation in the SMA was detected when the anode was placed over the M1, with a region-of-interest (ROI) analysis (Figure 3). The application of stimulation without motor task did not produce a detectable effect and neither did the inverse polarity (cathode over the M1) with or without finger tapping. Contrasting with these results, it was found that anodal tDCS simultaneously with grasp-release hand movements modulated activation at the primary somatosensory cortex (SM1) (Kwon and Jang, 2011). Subjects received anodal tDCS (left M1-CSR montage) for 2 min at 1 mA, resulting in increased cluster size and intensity related to the motor task.

A more complex pattern of tDCS induced changes in motor-related activation was described in the study by Stagg et al. (2009). As in the study previously described, tDCS was applied with a left M1-CSR montage with 1 mA intensity, but for a period of 10 min. The participants performed a serial reaction time task before and after stimulation. Whole-brain analysis showed task-related activity increased in the left M1, left PMd, and bilateral SMA when the anode was over the M1. With reversed polarity, bilateral M1, PMd, and posterior parietal cortex (PPC) changes were observed after. For a ROI analysis, the M1 and dorsal premotor cortex (PMd) of both hemispheres as well as the right frontopolar cortex (FPC) were selected and it was found that anodal stimulation was related with an increase in activation in the left M1 comparing to sham, whereas cathodal stimulation was associated with increased activation in the contralateral M1 and PMd. The FPC under the reference electrode did not show a stimulation effect, neither did a ROI at the primary visual cortex (V1) chosen as control, supporting a task specificity of the effects of tDCS. Furthermore, the authors found that cathodal stimulation led to an increase of functional connectivity of the M1 under the electrode with the contralateral M1 and PMd, whereas anodal stimulation did not alter connectivity of left M1 with other motor regions.

In the studies described so far, the position of the M1 electrode was determined by the motor representation of the hand area, detected using TMS, and the motor-task accordingly involved hand movements. Kim et al., 2012 applied anodal tDCS (cathode at CSR) over the leg representation on the right hemisphere for 15 min at 2 mA during rest, for 4 consecutive days. Whole-brain analysis revealed that after the fourth day activation elicited by toe flexion increased in the ipsilateral SMA and decreased in the contralateral M1, bilateral anterior cingulate gyri and right temporal and frontal region, in comparison with sham stimulation. Taken together, these results suggest a complex effect of tDCS, highly dependent on the stimulation paradigm and on the task being performed. This is not surprising, as physiological and behavioral studies have shown: timing between repetition of stimulation sessions as well as duration can be determinant (Monte-Silva et al., 2012), and the changes in the intensity of stimulation can even reverse effects of stimulation on cortical excitability (Batsikadze et al., 2013).

The after-effects of tRNS were also investigated using a finger-tapping task. After 4 min of stimulation (C3-CSR) at 1mA the extent of activation of the left sensorimotor cortex was decreased but no other significant changes were found (Chaieb et al., 2009). When tRNS was applied for 10 min during a visuomotor learning task (left M1-CSR montage), high-frequency tRNS caused a decrease in left frontal cortex activation, comparing with sham stimulation and a further decrease in bilateral frontal cortex and precuneous comparing with low-frequency tRNS (Saiote et al., 2013). This suggests a moderate effect of tRNS on BOLD response, however, it must be said that in this study, no changes due to tDCS were found. These are the only two studies combining tRNS and fMRI, and more are needed to understand how this technique is able to cause excitability (Terney et al., 2008) and behavioral (Fertonani et al., 2011) changes that have already been observed.

## Visual functions

To our knowledge, only one study investigated the effect of tDCS on visual perception. Combining cathodal tDCS of the right MT+ with a motion perception paradigm, (Antal et al., 2012a,b) observed increased activation of the MT+ after the stimulation (10 min). This effect was site specific, as no effect on the contralateral MT+ or V1 was found and the whole brain analysis did not detect significant changes. However, the results may not be task-specific as they did not depend on the difficulty level and there was not effect of stimulation at a behavioral level, in contrast with previous studies (Antal et al., 2004a,b). However, studies have shown that tDCS is also able to modulate cortical excitability of the visual cortex (Antal et al., 2003), contrast perception (Antal et al., 2001; Kraft et al., 2010) and visual evoked potentials (Antal et al., 2004c; Accornero et al., 2007). Therefore, it would be of interest to conduct more studies to characterize the tES modulation of visual function from primary to higher order level.

## tDCS and the resting brain

Intrinsic brain activity has been measured with BOLD-fMRI during rest to reveal a set of distinct groups of brain regions (networks) showing coherent activity at low-frequencies (0.01—0.1 Hz), which are functionally relevant, and comply with the underlying anatomy (Biswal et al., 1995; De Luca et al., 2006). This prompted the development of several techniques for analyzing resting-state fMRI data without the need to define an expected model of activation and instead based on functional connectivity measures (Van den Heuvel and Hulshoff Pol, 2010). Recent studies have used diverse approaches to analyze the wide-spread effects of tDCS on resting-state brain activity.

Polanía et al. (2011) investigated the effects of 10 min anodal tDCS at 1 mA over the M1 (left M1-CSR montage). They used an approach based on graph theory, which provides a theoretical framework for characterizing local and global properties of networks quantitatively (Stam and Reijneveld, 2007; Bullmore and Sporns, 2009). The simplest measure is the connectivity degree (K), which quantifies the number of connections of a voxel and was found to have increased in the left posterior cingulate cortex (PCC) and right dorsolateral prefrontal cortex (DLPFC). The characteristic path length (L) can provide information about the global character of connections, as it quantifies the minimum number of connections between two voxels, between nodes, thus measuring whether they are directly or indirectly connected. L was found to be increased in the left SM1, pointing toward a decrease of the direct distant connections with the rest of the brain (Polanía et al., 2011). To further characterize the observed changes in the PCC, right DLPFC, and left SM1, these regions were taken as seeds in a correlation analysis and it was found that the left PCC had increased connectivity with other regions from the default mode network (DMN). The DLPFC showed increased connectivity with the right anterior insula (part of executive control network). As for the left SM1, it showed increased connectivity with the left premotor and M1 as well as with the left SM1 and superior parietal cortex. Using other measure derived from graph theory, it was found that after 20 min of anodal stimulation of the right SM1 (C4-CSR montage), eigenvector centrality increased in the right prefrontal cortex, left middle temporal lobe, right fusiform, and middle temporal gyrus and bilateral cerebellum. Eigenvector centrality is a measure of the importance of a node within a network (Lohmann et al., 2010), according to which a high value indicates that a voxel is connected to other important nodes. Thus, this result provides further evidence of tDCS induced functional network reorganization (Sehm et al., 2012). This was compared to bilateral stimulation (anode C4—cathode C3) in which case eigenvector centrality increased in motor areas as the right M1, PMd and bilateral SMA as well bilateral prefrontal cortex. In this study, the dynamics of eigenvector centrality changes during the 20 min stimulation period were also investigated. Bilateral stimulation led to increases within the right M1/PMd (under the anode) but not by unilateral stimulation. Also in secondary motor areas, bilateral stimulation caused eigenvector centrality changes. Both montages caused changes in prefrontal cortex which may reflect an effect of the stimulation on the resting-state network and not specifically related to changes in excitability in the M1. In fact, bilateral vs. unilateral stimulation of the SM1 had previously been studied, although with opposite polarity. Voxel-count and signal intensity were found to be significantly higher after bilateral stimulation on the SM1 (anode left and cathode right SM1) comparing with unilateral stimulation (left SM1-CSR montage), for the short 1 min stimulation duration (Kwon and Jang, 2012).

Besides modulating the network between the M1 and other brain regions, it was found that tDCS interferes with the connections within the M1 itself (Polanía et al., 2012b). Cathodal tDCS over the M1 caused an increase of the clustering coefficient and anodal tDCS a decrease of the minimum path length in the hand/arm area. These changes were not due to alterations of the number of connections, since no significant changes were found regarding the connectivity degree caused by anodal or cathodal stimulation. Therefore, these results suggest that cathodal tDCS reinforces the local functional connections of the arm/hand area and anodal with the connection in other M1 areas. Interestingly, it was also found that the magnitude of changes depended on the baseline efficiency level.

Two studies have focused on the connectivity changes during rest observed after stimulation of the DLPFC. Keeser et al. (2011) applied anodal stimulation to the left DLPFC at 2 mA for 20 min and used independent component analysis (ICA) to identify 4 resting state networks: the DMN, the left and right fronto-parietal network (FPN) and the self-reference network (SRN) and found that, comparing with sham stimulation, there was an increase in connectivity within the DMN, left, and right FPN, which could be interpreted as an enhancement of the level of alertness. In the study by Peña-Gómez et al. (2012) used a right/left DLPFC—CSR montage and applied for 20 min at 2 mA intensity. Resting-state fMRI was measured for 10 min before and after stimulation and analyzed using ICA. The authors compared functional connectivity within the DMN, visual, and motor networks and found decreased functional connectivity when comparing the anterior and posterior regions of the DMN, accompanied by the detection of a new independent component (IC) similar to the anterior part of the DMN. Increased functional connectivity was found between prefrontal and parietal regions within a network showing negative correlation to the DMN (the anti-correlated network) together with the disappearance of parietal ICs, suggesting the merging of components. This effect was observed for both stimulation of the right and left DLPFC, but it was still considered as a specific effect, since no changes in functional connectivity of the motor and visual networks were found.

Evidence has also been provided regarding the influence of tDCS on cortico-subcortical functional connectivity using a seed-based approach (Polanía et al., 2012a). The nucleus accumbens, caudate, putamen, and thalamus were taken as seeds in a multiple regression analysis during resting state, comparing before and after 10 min of anodal or cathodal stimulation over the M1. After anodal tDCS, connectivity between the left thalamus and M1 increased, as well as between the left caudate and superior parietal lobule. Also, connectivity decreased between the left caudate and the PCC. Polarity reversal led to decreased connectivity between the right putamen and left M1 and between the right thalamus and left superior frontal gyrus.

## The simultaneous effect of tDCS on higher cognitive functions and on brain activity

The tES-induced after-effects are not only limited to the motor and visual functions or on resting state activity but considerable evidence emerged for a reliable behavioral effect of tES on the cognitive functions (for a recent review see Kuo and Nitsche, 2012). The investigation of the effect of tDCS on cognition combined with fMRI is a particularly interesting approach, since it provides unique information about the tES-induced behavioral changes accompanied by the neural alternations.

Only two studies have directly investigated the effect of stimulation on cognitive functions with the combination of neuroimaging, and both studies used the concurrent application of tDCS and fMRI. Holland et al. (2011) targeted the left inferior frontal cortex (IFC) with anodal tDCS (cathodal electrode over the CSR) and measured neural and behavioral changes in a picture-naming paradigm. The stimulation was 20 min long with an intensity of 2.0 mA resulting in a current density of 0.057 mA/cm^2^. They found a significant facilitation in picture naming performance in the active tDCS condition, compared to the sham tDCS condition (using a cross-over design). These behavioral findings were accompanied by a significant reduction of the BOLD response in the left IFC, including the Broca's area. In a more recent study, (Meinzer et al., 2012) investigated the effect of anodal tDCS on a semantic word generation task by stimulating again the left IFC (cathodal over the CSR). Ten minutes long anodal tDCS over the Broca's area at 1.0 mA (with a current density of 0.029 mA/cm^2^ under the anodal electrode) improved semantic word generation performance, by increasing the number of the correct answers during active vs. sham tDCS. Interestingly, similar to the findings of Holland and colleagues, this behavioral enhancement was also associated with a selective reduction of the BOLD-response in the left ventral inferior frontal gyrus (vIFG) and with an increased language network connectivity evidenced by a graph-based eigenvector centrality mapping data analysis approach, this latter result suggesting an enhanced efficacy in information processing at the neural network level.

At the present, it is difficult to draw a conclusion from these two studies. The experimental evidence so far suggests that in both cases the tDCS-induced changes in higher cognitive functions were associated with reduced BOLD activity in highly-specific task-related brain regions. These findings are converging despite the strikingly different stimulation protocols concerning the stimulation duration and the intensity. A remaining issue for future studies would be providing further evidences for the relationship between altered cognition and the associated neural changes in the functionally relevant brain regions.

## Summary

The aim of this review was to summarize results so far obtained by combining tES and fMRI. Taken together, the studies presented here provide valuable insight to the potential of tES, as a tool to modulate brain activity. Modeling studies estimate a spatially wide distribution of the electric field induced during tDCS (e.g., Bikson et al., 2012), which seems to be confirmed by the whole brain effects of the stimulation on brain activity. However, it is especially interesting that the findings gathered from tDCS-fMRI up to this point, do not show an unspecific change in the neuronal activity, but are found in rather functionally relevant and task-related brain regions. Therefore, despite tDCS and generally tES techniques being considered as a non-focal NiBS methods (compared to TMS for example), it can be that the effects of stimulation are functionally focal. However, it is not clear how much of the dispersion of tES-induced changes is mediated by the functional state of the brain or by the wide reach of the induced electric field. Efforts have been made to improve focality of tDCS so as to enable controlled targeting of the stimulation and a setup for high-definition tDCS has been developed (Datta et al., 2010). Therefore, it would be interesting to see how the focality of physiological effects that has been achieved (Edwards et al., 2013) translates to whole-brain activity and its interplay with functional brain state.

Other tES modalities, namely tACS and tRNS are not expected to act by the same mechanisms as tDCS, not only regarding local physiological changes, but also at a functional and network level. To our knowledge, no study combined fMRI with tACS and only two studies combined it with tRNS (Chaieb et al., 2009). The possibility of differentially interact with rhythmic brain activity is very enticing and calls for more studies regarding these techniques, as knowledge of their effects is quite limited and can be complemented by fMRI.

What also remains to be further elucidated at a whole-brain level, is the temporal evolution of the tES-induced changes, not only in terms of duration of after-effects, but also regarding the influence of protocols and repeated stimulation sessions. As a clinical tool, tDCS has found application in several neurological conditions (Nitsche et al., 2008), such as stroke, depression and Parkinson's disease with a diversity of stimulation paradigms, stressing the importance of understanding the specific effects and dependence of tES on the parameters chosen for its application. Furthermore, the impact of tES on functional brain networks already altered by disease is yet to be studied and related to current clinical findings, as well as compared with that of healthy subjects.

### Conflict of interest statement

The authors declare that the research was conducted in the absence of any commercial or financial relationships that could be construed as a potential conflict of interest.
